# Supplementary figures and images for: Co-expressed microRNAs associated with elevated psychometabolic risk phenotype in women during midlife
Source: Front Endocrinol (Lausanne). 2026 Jun 23;17:1816057. doi: 10.3389/fendo.2026.1816057 (PMC13337358; doi:10.3389/fendo.2026.1816057)

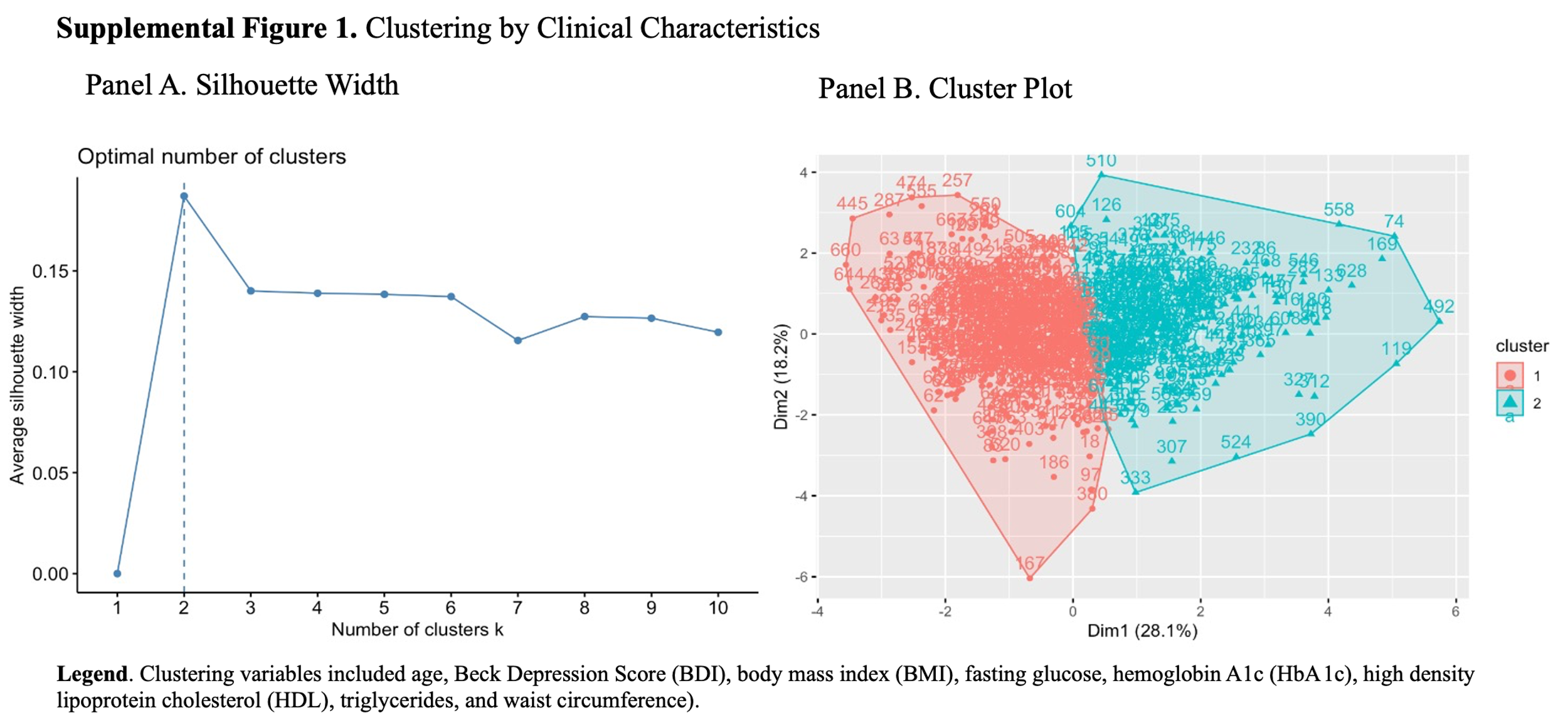

Supplement: Supplementary file 1 [file DataSheet1.docx]
